# Indents

**Published:** 1917-05

**Authors:** 


### INDENTS

☞ **Brief Articles of Technical or Scientific Value will receive notice and comment. Contributions invited.**

*Inlay Impression* To secure a correct impression or wax model for a molar M. O. D. cavity, first force the softened wax into the cavity so that it fills both interproximal spaces. Then trim away most of the excess wax except in the interproximal spaces. Then soften the occlusal surface and have the patient occlude the teeth firmly. Retrim the excess wax from the occlusal and marginal surfaces. Now trim the proximal surfaces so that a very thin matrix can be placed between the teeth and with thin right and left burnishers, applied over the matrix, force the wax into the cavity to a close adoption with the cervical and lateral cavity walls, being careful to preserve the general tooth form and as good mesiodistal contacts as possible. In doing this the wax should be held in the cavity firmly by placing the finger on the occlusal surface, while the burnishers are passed in from the buccal and lingual surfaces, working the wax always toward the margins. The matrix is then removed and the careful trimming and finishing usually done complete the mould wax. Remove and add a little hot wax to restore the contact points. If any of the wax at the margins breaks away, don't try to restore it by adding hot wax but replace in the cavity and insert the blade of warm burnisher into the wax slightly away from the margin and force the wax out against the border, and then fill the instrument mark with hot wax and refinish the surface. Wax mould made in this way will give accurate fitting castings and the process is simple and definite technically.—F. S. Meyer in April *Items of Interest* Round Table.

*Dental Maternity Clinic*

A dental clinic has recently been opened for the treatment of expectant and nursing mothers, and for children under school age, in connection with the Maternity and Infant Welfare Center, at Wealdstone and that of the Harrow Council. The successful establishment of this clinic is in large degree the result of the endeavors of Dr. Barbara Tchaykoysky, who has lost no occasion to draw the attention of parents and authorities to the importance of dental treatment for mothers and early care of children's teeth.—*Dental Record.*

*Canadian Army Dental Statistics*

From October first to December thirty-first, 1916, the Overseas Dental Corps in England reports the following statistics: Fillings made, 52,253; treatments, 13,723; dentures, 10,276; prophylaxis, 4,056; extractions, 59,676; pulps devitalized, 4,957, a total of 144,943 operations of all kinds made in three months. This statement will indicate in no uncertain terms the value of the dental service branch in the army.

*Zinc-Oxid-Eugenol Paste as a Root Filling*

When the root canal is properly cleaned and enlarged, first with Kerr's broaches and later with a Gates-Glidden drill, and dried out, the writer employs the following method for filling the canal:

Three parts of beeswax and three parts of lanolin are melted together with one part of thymol and one part of eugenol. This will form a thick paste, and a little of this is placed on a spatula, dipped into a forty per cent. solution of formalin, then mixed well together on a slab with zinc oxide until a very thick paste is produced. From this paste cones are formed and one of them is placed on the end of the canal plugger and introduced into the enlarged and cleaned root canal, and pushed down until it fills the canal completely. A small ball of Hill's stopping is put on top of this, and the filling is pushed down until the patient feels a slight pain—the proof that the filling has reached the apex.

This paste gets very hard. I have found it quite unchanged after it had been left in for two years, and without any odor whatever.

This paste may be used with very good results for filling children's teeth. The preparation is as mentioned above, only instead of mixing the paste with cotton, asbestos fibres are used. The filling may be left in for three or four years.

In cases where it is difficult to see whether the pulp has been exposed or not, especially in young teeth, I cover the cavity with this paste, and leave it in for three or four months. If the patient suffers any pain from this treatment, the pulp must of course be removed; but generally the result is that the pulp contracts, and afterward the cavity may be filled without the pulp having been removed. I leave a little of the paste in the part of the cavity nearest to the pulp.—E. A. Bruun in April *Cosmos.*

*Chlorin as a Disinfectant* Of all the known chemic disinfectants, chlorin, freshly prepared, and in the presence of moisture and a suitable temperature, possesses the greatest known germicidal power. Chlorin forms the active constituent of Labarraque's solution, Javelle water, antiformin, otherwise known as dental radicin, electrozone, or dental meditrina, and of the antiseptic solution so successfully employed at present in the English and French war hospitals according to Dakin's formula by the Carrel method, which latter preparation is now advertised abroad as eusol, and in our country in the form of a salt, as chlorazene. The great success obtained with Dakin's solution rests primarily upon the fact that a fresh preparation according to a specific

(Carrel) method is employed, which, while acting deleteriously on the germs, does little harm to the tissue cells, as it is a nonirritating isotonic wound antiseptic. All of these solutions have sporadically come to prominence in the past. While the laboratory reports, as far as the germicidal power of these solutions is concerned, appear to be highly satisfactory, practical application does not bear out the enthusiasm. It should be borne in mind that most of these preparations are irritating, and that chlorin solutions are liable compounds. All of these enumerated solutions lose their activity within a week or two, hence the disappointing results when commercial stock preparations are employed.

The rationale of sterilizing an infected wound surface by the Carrel method is based on the following conception: "To render an infected wound sterile it is necessary to employ a suitable antiseptic in such a manner that the chosen antiseptic comes into contact with every portion of the wound, that the antiseptic is maintained in a suitable concentration throughout the entire wound, and that this constant strength is maintained for a prolonged period. If these conditions are fulfilled, every wound will show its response to the treatment by the diminution and disappearance of its microorganisms." The ionization of a one per cent. sodium chlorid solution by means of the galvanic current within a root canal furnishes free chlorin *in status nascendi* in the presence of moisture and body temperature which is most active, and admirably suited for our purposes. The free chlorin sterilizes the walls of the root canal (see below) and bleaches the discolored dentin. Incidentally, the ionization of the salt water furnishes an appreciable amount of hydrochloric acid which acts as a superficial solvent of tooth structure, and thereby enlarges the root canal. The two chemicals, according to Lehmann-Zierler, are present in the proportion of five parts chlorin to three parts hydrochloric acid. The small quantities of sodium compound which are formed within the vicinity of the negative pole are taken care of by the tissue fluids.—H. Prinz in April *Cosmos.*

*Should We Extract All Diseased Deciduous Teeth?* These teeth at five years and seven years, the deciduous molars, should be retained in the mouth if they can be retained in a healthy condition; and ordinarily they can, even if there is an abscess—that is, a sinus opening—because there is no class of cases that I know of that will respond so quickly and so easily to treatment of that condition as will those deciduous molars. And I think that if an orthodontist were talking upon this subject, he would ask you to keep them there if at all possible in order to maintain the space. If these teeth are extracted too early, you are very likely to invite an irregularity. If you will take those little children and treat them carefully, you can as a rule clear up all such conditions. I am not talking on the technical part of this question, simply on the general aspect of it.

I would not keep an abscessed tooth in a child's mouth that could not be cured, but I am going to announce today that I have more faith at the present time in curing those abscesses, both in deciduous and in permanent teeth, than I had a year ago. In this connection, I may say that not all the abscessed teeth that come to my office are extracted. —*Dental Review.*

*A Peculiar Case* Dr. Margaret Ormiston, a school medical inspector under the Cheshire Education Committee, has recorded a very interesting case of hemophilia met with in a boy twelve years of age. He was recognized as a "bleeder" when quite small and due precautions were taken; only on one occasion, when he was about eight or nine years old, was any trouble experienced, and that was after he had a tooth extracted. He then lost a large quantity of blood and had to have medical attention. There are two interesting points about the case, the first being that the slightest push, such as one might playfully give to a child while telling him to "hurry up," produced extensive ecchymoses, one occasion being recorded when the exact shape of a hand was found on the boy's back after he had been gently pushed on by a relative. Whenever he fell or knocked himself in play he did not have a bruise as other children have, but an extensive coalblack patch appeared. He suffers from defective sight, and his spectacles were frequently taken from him during play in order to prevent their being broken and his face possibly being cut. The other unusual feature of the case is that just before he was twelve all trace of this disease vanished. He no longer bleeds to excess and blows and knocks produce only an ordinary bruise. There is no history of hemophilia in either his father's or mother's families.—*Dental Record.*

*Treatment of Baldness and Dandruff* Chas. J. White, of Boston, professor of dermatology at Harvard University, after an experience with 704 cases of alopecia and seborrhea of the scalp, and finding that women are more often affected than men, gives it as his opinion (*Jour. Amer. Med. Asso.*) that of the various lotions, ointments, and soaps, besides internal medication and massage tried by him, the best results were obtained with a preparation composed as follows:

Hydrargyri chlorodi corrosivi........grs. 4
Euresol pro capillis..................drs. 2
Spiritus formicarum...................oz. 1
Olei ricini................(dr. 1 to) drs. 3
Alcoholis, q. s. ad...................ozs. 8

Label: Wash for scalp. (Poison.) Apply in the morning.

The active ingredient of this lotion is euresol (mono-acetate of resorcin). With this, Doctor White obtained gratifying results in many most intractable cases.—*Clinical Medicine.*

